# Electric field-directed migration of mesenchymal stem cells enhances their therapeutic potential on cisplatin-induced acute nephrotoxicity in rats

**DOI:** 10.1007/s00210-022-02380-7

**Published:** 2023-01-14

**Authors:** Shaimaa A. Abdelrahman, Nermin Raafat, Ghadeer M. M. Abdelaal, Sara M. Abdel Aal

**Affiliations:** 1 Medical Histology & Cell Biology Department, Faculty of Medicine, Zagazig University, Zagazig, Egypt; 2 Medical Biochemistry and Molecular Biology Department, Faculty of Medicine, Zagazig University, Zagazig, Egypt; 3 Forensic Medicine & Clinical Toxicology Department, Faculty of Medicine, Zagazig University, Zagazig, Egypt

**Keywords:** Electric field, Mesenchymal stem cells, Cisplatin, Acute kidney injury, Rats

## Abstract

Cisplatin is widely used as an anti-neoplastic agent but is limited by its nephrotoxicity. The use of mesenchymal stem cells (MSCs) for the management of acute kidney injury (AKI) represents a new era in treatment but effective homing of administered cells is needed. This study aimed to investigate the effect of bone marrow-derived mesenchymal stem cells (BM-MSCs) on cisplatin-induced AKI in rats after directed migration by electric field (EF). Forty-eight adult male albino rats were equally classified into four groups: control, cisplatin-treated, cisplatin plus BM-MSCs, and cisplatin plus BM-MSCs exposed to EF. Serum levels of IL-10 and TNF-α were measured by ELISA. Quantitative real-time PCR analysis for gene expression of Bcl2, Bax, caspase-3, and caspase-8 was measured. Hematoxylin and eosin (H&E) staining, periodic acid Schiff staining, and immunohistochemical analysis were also done. MSC-treated groups showed improvement of kidney function; increased serum levels of IL-10 and decreased levels of TNF-α; and increased mRNA expression of Bcl2 and decreased expression of Bax, caspase-3, and caspase-8 proteins comparable to the cisplatin-injured group. EF application increased MSCs homing with significant decrease in serum urea level and caspase-3 gene expression together with significant increase in Bcl2 expression than occurred in the MSCs group. Restoration of normal kidney histomorphology with significant decrease in immunohistochemical expression of caspase-3 protein was observed in the BM-MSCs plus EF group compared to the BM-MSCs group. EF stimulation enhanced the MSCs homing and improved their therapeutic potential on acute cisplatin nephrotoxicity.

## Introduction

Acute kidney injury (AKI) is a clinical illness characterized by a sudden loss of renal excretory function, accumulation of metabolic acids and nitrogen metabolism products, elevated potassium, and phosphate levels in the blood (Hsu et al. 2007). Clinically, ischemia-reperfusion (I/R), antibiotics such as gentamicin (Muthuraman et al. 2011), anticancer agents such as cisplatin and ifosfamide (Izuwa et al. 2009; Zhang and Lu 2007), radio contrast media, non-steroidal anti-inflammatory drugs (NSAIDS), osmotic changes, and folic acid (Efrati et al. 2007; Singh et al. 2012), all contribute to the multifactorial and complex etiology of AKI.

Cisplatin (cis-diamine dichloro platinum (II), Csp) is a common anti-neoplastic drug used to treat a variety of malignancies, including germ cell tumors, lymphomas, sarcomas, and carcinomas (Atessahin et al. 2005). It works well and consistently as a chemotherapeutic treatment for solid tumors. Due to adverse effects such as nephrotoxicity, it cannot be used in high dosages or for prolonged periods of time during a treatment plan (Leite et al. 2021). After receiving a single dosage of cisplatin, 25–35% of patients experience nephrotoxicity (Lee et al. 2009). At 72 h after cisplatin administration, the highest concentration of cisplatin was found in the mitochondria (37%), followed by cytosol (27%), nuclei (22%), and microsomes (14%) (Levi et al. 1980). Cisplatin causes cytotoxic lesions in cancers and other proliferating cells after being bioactivated by hydrolytic processes after it reaches the cell. These aqua species are more reactive than the neutral molecule (Humanes et al. 2012). Many recent studies emphasized the toxic effects of cisplatin on the kidney at the molecular levels through induction of inflammatory pathways, apoptosis, and oxidative stress (Anbar et al. 2022; Wang et al. 2022; Xie et al. 2022).

The efficiency of MSC-based therapy has been demonstrated in recent studies and clinical trials as a viable alternative method in the treatment of various traumatic, toxic, and pathological disorders in many body organs, such as cardiac illnesses (Müller et al. 2018), hepatic diseases (Yang et al. 2020), muscular dystrophies (Biressi et al. 2020), articular cartilage damage (Vahedi et al. 2021), renal toxicity (Wu et al. 2021), spinal cord injuries (Huang et al. 2021), traumatic brain injuries (Heile and Brinker 2022), and diabetic wound healing (Ho et al. 2022).

Mesenchymal stem cells (MSCs) were used in this work to try to counteract the nephrotoxic effects of cisplatin. MSCs are adult non-hematopoietic progenitor cells that have been isolated from a variety of organs, including the placenta, bone marrow, and adipose tissue. MSCs are known to attach to plastic and exhibit certain cell surface markers. They could differentiate in vitro into mesenchymal lineages such as chondroblasts, adipocytes, and osteoblasts (Dominici et al. 2006). Since MSCs emit a variety of cytokines and paracrine factors, including anti-inflammatory neurotrophic, angiogenic, immunomodulatory, antifibrotic, antiapoptotic, and survival factors, they are helpful in tissue healing (Caplan and Correa 2011; Larsen and Lewis 2011; Mundra et al. 2013). According to Burks et al. (2015), treatment times for established AKI experiments were derived from the natural history of cisplatin-induced AKI. In their study, MSCs were injected on day 3, after renal function declined.

Transferring mitochondria has a significant part in accelerating tissue healing. A cell’s damaged mitochondria can be replaced by healthy mitochondria from MSCs, restoring the cell’s normal biological function and ensuring cell survival. Different structures, such as gap junctions (GJ), tunnel nanotubes, extracellular micro-vesicles, and cell fusion, mediate the transport of mitochondria. Connexin 43 (Cx43), the primary GJ protein, allows wounded cells and MSCs to establish GJ channels. Vesicles containing mitochondria are then released from MSCs to reach injured cells and are endocytosed (Zhang et al. 2022).

In non-injured states, intravenous MSCs tend to migrate to the bone marrow (Wynn et al. 2004). After an injury, MSCs gravitate towards the area of inflammation where they traverse the inflamed endothelium bed (Herrera et al. 2007). MSCs can infiltrate wounded tissue through a variety of interactions between chemokines released by the injured tissue and chemokine receptors expressed by MSCs (Ponte et al. 2007). Migration of BM-MSCs was discovered to be mediated by interactions between stromal cell-derived factor-1a (SDF-1a) and its chemotactic receptor C-X-C chemokine receptor type 4 (CXCR4) (Ries et al. 2007).

The efficiency of cells to integrate and function in the target tissues is significantly improved by efficient homing of the transplanted cells (Naderi-Meshkin et al. 2015). It has long been known that the electric field (EF) is a crucial cue that directs the sustained migration of numerous distinct cell types. In numerous cell populations from mammalian, amphibian, and fish species, galvanotaxis, or the directional migration of cells in an EF, has been discovered (Iwasa et al. 2017).

EF application was discovered to improve MSC homing (Soliman et al. 2017) as BM-MSCs have been migrated strongly towards the anode in response to a modest application of EF. In addition, physiological levels of EF stimulation showed no effect on the phenotypic, osteogenic potential, or cell senescence of MSCs according to Zhao et al. (2011). It is known that EF causes the brain progenitor cells to migrate quickly and purposefully (Babona-Pilipos et al. 2011; Feng et al. 2012). A clinical trial in spinal cord injury revealed significant recovery of the injured area after exposure to the weak EF (Tator 2005). Another study showed that physiological EFs aided neural stem/progenitor cell migration towards the cathode (Li et al. 2008).

From all the above, the present study was conducted to delineate in more details the biochemical and histomorphological effects of BM-MSCs treatment on AKI model induced by cisplatin in adult male albino rats and to evaluate MSCs homing and efficacy after EF stimulation.

## Materials and methods

### Site of study

- This study was carried out in the Stem Cell Research Unit in Zagazig Medical and Scientific Research Centre in collaboration with Histology and Cell Biology Department, Faculty of Medicine, Zagazig University.
- Photography was performed at Image Analysis Unit, Histology and Cell Biology Department, Faculty of Medicine, Zagazig, Egypt.
- Flow cytometric characterization of BMSCs was performed at Clinical Pathology Department, Faculty of Medicine, Zagazig University.
- Morphometrical analysis was done in Pathology Department, Faculty of Dentistry, Cairo University, Cairo, Egypt.

### Animal model and experimental design

- The study used 48 adult male albino rats (180–200 g) from the Animal House, Faculty of Medicine, Zagazig University. Throughout the course of the experiment, rats had unlimited access to food and drink while living in a room-temperature environment with 12-h light/dark cycles. To eliminate sex differences in this study, male rats were used as estrogen itself enhances cisplatin-induced nephrotoxicity (Leite et al. 2021). All experimental procedures were performed in accordance with the guidelines of the Institutional Animal Care and Use Committee (IACUC) approved by the Faculty of Medicine, Zagazig University (Approval number: ZU-IACUC/3/F/143/2022).
- Cisplatin (Csp, CAS No. 15663–27-1) was purchased from Sigma-Aldrich Co. (St. Louis, MO). It was dissolved in sterilized normal saline and prepared immediately before the treatment. Csp (7 mg/kg) was injected to rats by intraperitoneal route of injection on test day 0. The application volume of Csp was 2 mL/kg body weight (Lee et al. 2017). Food and water were withheld from rats for 12 h before cisplatin injection until after cisplatin injection (Burks et al. 2015).

**Fig. 1:**
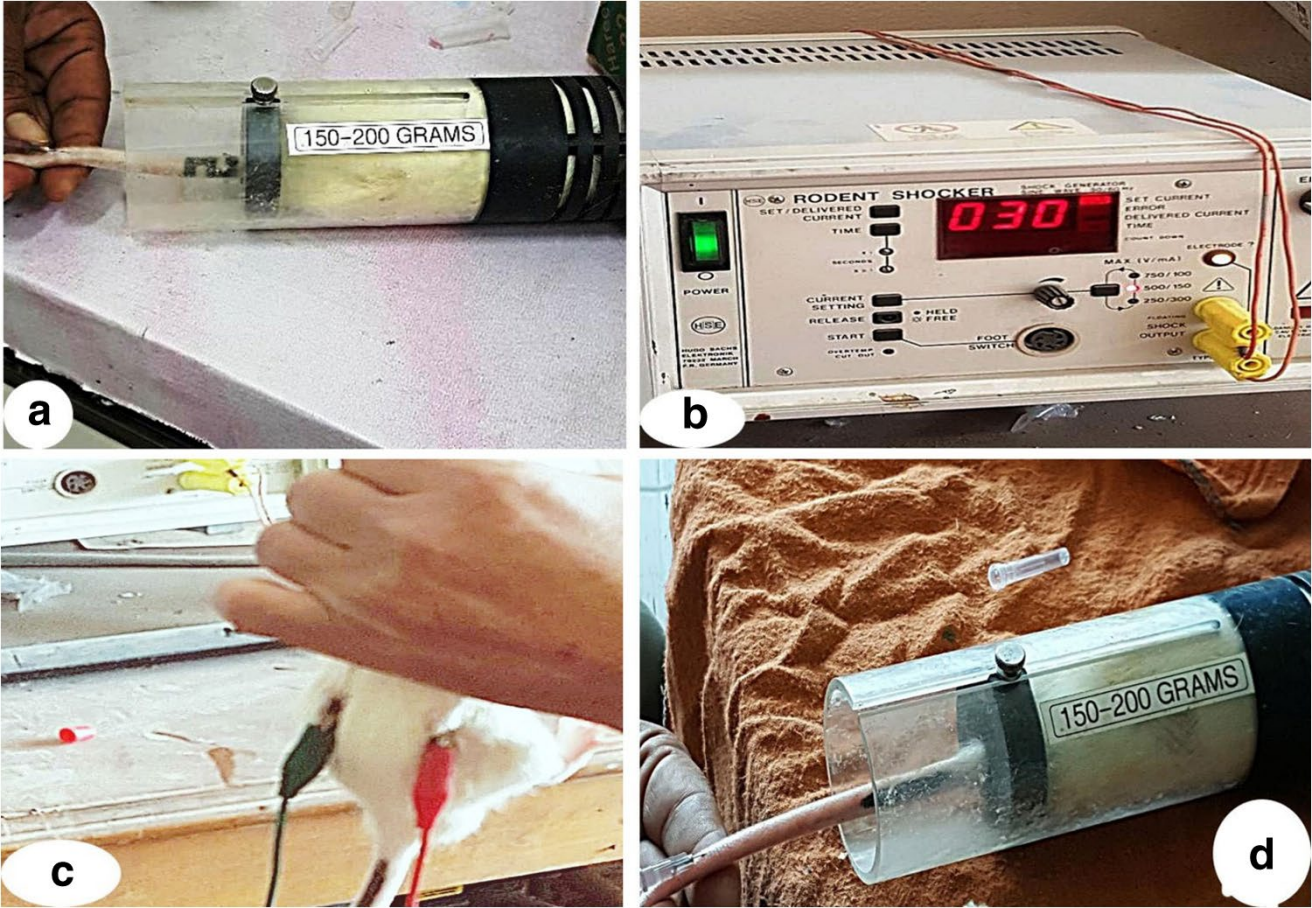
Showing **a** intravenous injection of BM-MSCs (1 × 10^6^ cells/mL), **b** rodent shocker adjusted at 30 mV/mm, **c** rats were exposed to an EF (30 mV/mm) on their back for 10 min, **d** intravenous injection of BM-MSCs (1 × 10^6^ cells/mL) after EF stimulation

The animals were classified into four groups:

- *Group I (control)*: included twelve rats that were equally subdivided into two subgroups (six rats each).
  - *Subgroup Ia (negative control group)*: each rat received only regular diet and tap water to measure the basic parameters.
  - *Subgroup Ib (vehicle-control group)*: rats received a single intraperitoneal dose of sterilized normal saline in an equivalent volume to that in cisplatin (2 mL/kg body weight).
  *Group II (cisplatin-treated)*: included 12 rats received a single intraperitoneal injection of cisplatin at a dose of 7 mg/kg on test day 0 (Lee et al. 2017).
- *Group III (cisplatin plus BM-MSCs)*: included 12 rats, each rat received a single intraperitoneal injection of cisplatin as group II followed by intravenous injection of BM-MSCs at a dose of 10^6^ cells suspended in 0.5 mL phosphate buffered saline (PBS) on day 3 after AKI induction with cisplatin (Fig. 1a) (Burks et al. 2015; Soliman et al. 2017).
- *Group IV (cisplatin plus BM-MSCs exposed to EF)*: included 12 rats with AKI received intravenous BM-MSCs as group III and were exposed to an EF (30 mV/mm) on their back for 10 min (Fig. 1b–d) (Soliman et al. 2017).

### Sampling

All animals were euthanized under full anesthesia by intraperitoneal injection of sodium thiopental (25 mg/kg) (Kara et al. 2014) on day 7 post-cisplatin (Burks et al. 2015). Venous blood samples were collected from animals by means of micro-capillary glass tubes from the retro-orbital plexus, then kidney tissue samples were taken for biochemical and histological studies, respectively.

### Experimental procedures

#### Preparation of bone marrow derived-mesenchymal stem cells (BM-MSCs)

Male albino 6-week-old rats’ tibiae and femurs were flushed with Dulbecco’s modified Eagle’s medium (DMEM) to extract the bone marrow, supplemented with 10% fetal bovine serum. Nucleated cells were isolated with a density gradient and re-suspended in complete culture medium supplemented with 1% penicillin-streptomycin. Cells were incubated at 37 °C in 5% humidified CO_2_ for 12–14 days, until formation of large colonies (80–90% confluence). The culture was washed with PBS and released with 0.25% trypsin in 1 mL EDTA (5 min at 37 °C). After centrifugation, cells were re-incubated in 50 cm^2^ culture flask (Falcon) with serum-supplemented medium (Raafat et al. 2015; Abdelrahman et al. 2018). MSCs in culture were identified by their fusiform shape and adhesiveness (Rochefort et al. 2005). The trypan blue dye exclusion test was used to determine the vitality of the cells. This technique is based on the idea that while dead cells do absorb some colors, healthy cells do not (Haasters et al. 2009).

#### Labeling of stem cells with Paul Karl Horan 26 (PKH26)

BM-MSCs were harvested during the 2nd passage and were labeled with PKH26 red Fluorescent Cell Linker Kit (Sigma, St. Louis, Missouri, USA) prior to rat injection (Haas et al. 2000). Cells were centrifuged and washed twice in serum-free medium. Cells were pelleted and suspended in dye solution.

#### Characterizations of rat BM-MSCs by flow cytometry

**Fig. 2:**
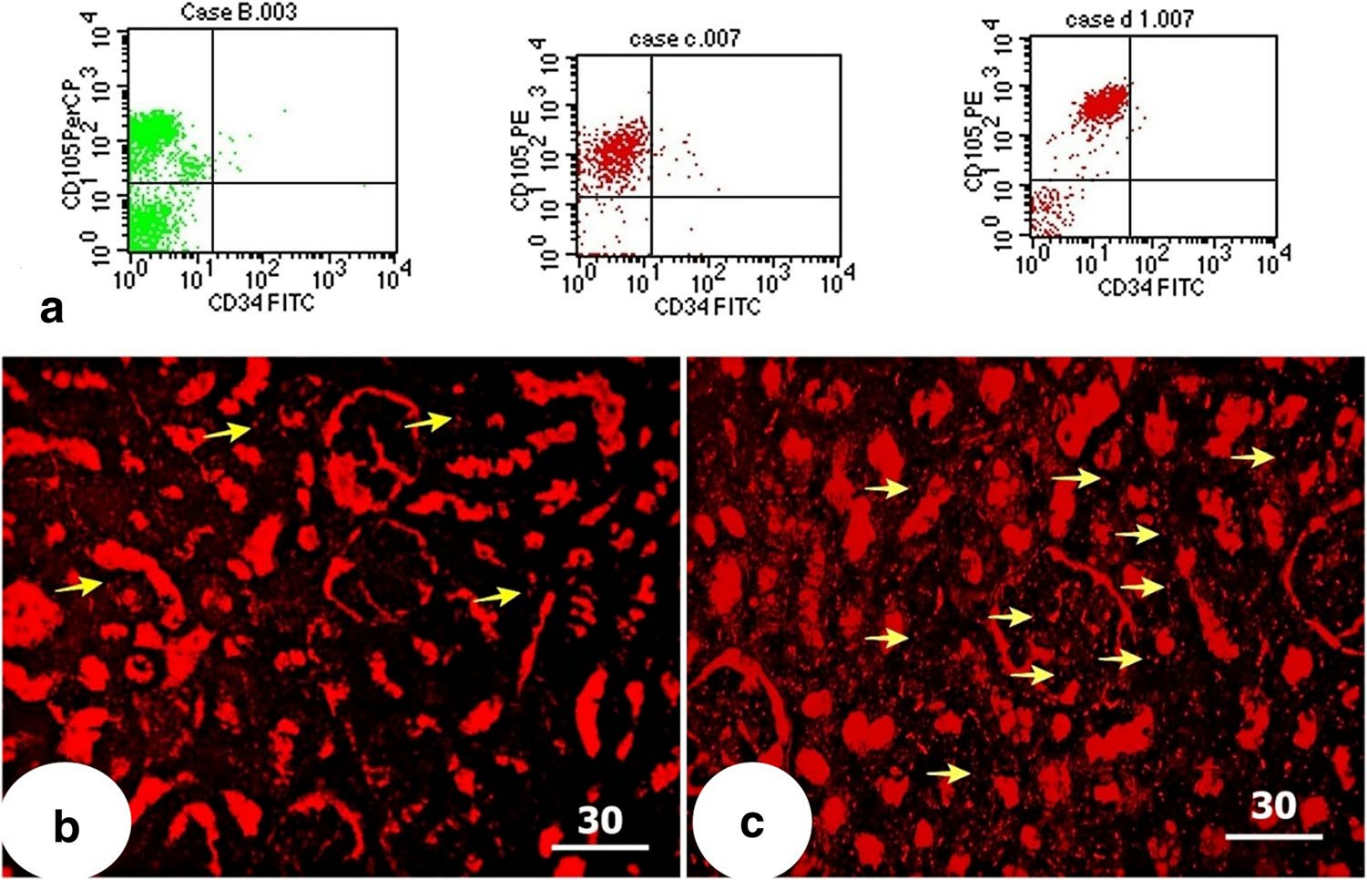
**a** Flow cytometric analysis of the cell surface markers showing positive expression of CD105 (mesenchymal stem cell surface marker) and negative expression of CD34 (hematopoietic stem cell marker). **b** and **c** PKH26-labeled MSCs appearing as bright dots (yellow arrows) in the MSC-treated (group III) and MSC + EF (group IV), respectively (fluorescent microscope × 200, scale bar: 50 μm)

BM-MSCs were characterized by their adhesiveness and fusiform, star, or spindle shape. Flow cytometric characterization of BMSCs showed positive expression of CD105 surface marker (PE labeled), while the majority of cells were negative for CD34 surface marker expression (FITC labeled) (Barry et al. 1999; Conget and Minguell 1999) as shown in Fig. 2a.

#### Detection of homing of stem cells

Kidney tissue was examined with a fluorescent microscope (Olympus BX50F4, No. 7M03285, Tokyo, Japan) to detect and trace the cells stained with PKH26.

### Biochemical analysis

#### Kidney function assessment

Blood urea nitrogen (BUN) and serum creatinine level were determined spectrophometrically using kits provided by Elitech (France). Serum urea was determined according to the method of Fawcett and Scott (1960), and serum creatinine concentration was measured according to the method of Henry et al. (1974).

#### Enzyme-linked immune-sorbent assay (ELISA)

Post-transplantation, serum from all rats was isolated and assayed for interleukin 10 (IL-10) with accession number P29456 and tumor necrosis factor α (TNF-α) with accession number P16599 production with ELISA Quantitation kit (Raybiotech, Georgia, USA) according to the manufacturer’s recommendation.

#### Quantitative real-time PCR analysis

Real-time PCR of target gene copy numbers in relation to glyceraldehyde-3-phosphate dehydrogenase (GAPDH) transcripts was carried out using individual primers mentioned in Table 1. Gene expression levels were quantified using a SYBR Green kit (Qiagen, Germany). Data were analyzed using MxPro quantitative PCR Software (Agilent technologies). Table 1 lists the oligonucleotide primer sequences used for GAPDH, VEGF, caspase-3, caspase-9, Bax, Bcl2, and IL-6 (Hua et al. 2015; Li et al. 2014).

**Table 1:** Primer’s sequence of GAPDH, caspase-3, caspase-8, Bax, and Bcl2 genes

Target	Sequence
GAPDH	F: 5′- TGACATCAAGAAGGTGGTGA-3′ R: 5′- TCATACCAGGAAATGAGCTT-3′
Caspase-3	F: 5′-AATTCAAGGGACGGGTCATG-3′ R: 5′-TGACACAATACACGGGATCTG -3′
Caspase-8	F: 5′-GATGAGGCAGACTTTCTGCT -3′ R: 5′-CATAGTTCACGCCAGTCAGGAT -3′
Bax	F: 5′-GGCGAATTGGAGATGAACTG -3′ R: 5′-TGCCATCAGCAAACATGTCA -3′
Bcl_2_	F: 5′-ATCCAGGATAACGGAGGCTG-3′ R: 5′-CAGGTATGCACCCAGAGTGA -3′

The PCR was performed in 25 μl containing 12.5 μl 2× QuantiFast SYBR Green PCR Master Mix, 1 μM of each primer, and 5 μl cDNA with the following conditions: 95 °C for 5 min, then 40 cycles at 95 °C for 10 s, and combined annealing and extension 60 °C for 30 s. All kits were supplied by (QIAGEN, Valencia, CA, USA). Samples were assessed in duplicates to make sure the reproducibility and accuracy of the obtained results.

#### Histological study

For light microscopic examination, tissue samples were fixed in 10% neutral formalin prior to sectioning into 5-μm-thick sections. Sections were stained with hematoxylin and eosin (H&E) to evaluate general tissue morphology (Bancroft 2013). PAS reaction was applied for histochemical detection of carbohydrates (Bancroft and Gamble 2008).

#### Immunohistochemical study

The streptavidin-biotin complex immunoperoxidase technique was used for the immunohistochemical detection of the anti-caspase-3 protein. On charged slides, serial portions of specimens with paraffin embedding were deparaffinized. After blocking endogenous peroxidase for 30 min with 0.1% hydrogen peroxide, the sections were treated with the primary antibody (polyclonal anti-caspase-3, produced in rabbit, IgG fraction of antiserum-Catalog Number C9598, 1:500 dilution, purchased from Sigma-Aldrich 3050 Spruce Street, St. louis, MO 63103 USA). The primary antibody was incubated for 20 min at room temperature. After several washes with PBS, primary antibodies were detected by incubation with biotinylated anti-rabbit antibodies (versal kits, Zymed laboratories) for 30 min at room temperature. Thereafter, all sections were incubated with the streptavidin-biotin peroxidase complex for 30 min at room temperature. After washing with PBS, reactions were visualized with 3′, 3Regular-diaminobenzidinetetrahydrochloride (DAB — Sigma-Aldrich Chemical Co., St. Louis, USA) used as chromogen to visualize antibody binding. The sections were counterstained with Mayer’s hematoxylin, dehydrated, and mounted. For negative control sections, the primary antibody was replaced with PBS (Ramos-Vara et al. 2008).

#### Histo-morphometric analysis

Histo-morphometric analysis of the area percent (area %) of PAS, caspase-3 expression, and the number of fluorescent-labeled MSCs was done at the Image Analysis Unit of Pathology Department, Faculty of Dentistry, Cairo University, Egypt using Leica Qwin 500 image analyzer computer system (Leica Ltd., Cambridge, UK). They were measured using the interactive measure menu. Five slides of five different specimens were examined for each group. Ten nonoverlapping fields were measured of each sample from each group at a magnification of ×400.

### Statistical analysis

The data obtained for all groups were expressed as mean ± standard deviations (SD) and subjected to statistical analysis using one-way analysis of variance (ANOVA) test for comparison between the different studied groups (more than two groups). Least significant difference (LSD) test was also done to find significance in between groups (Dean et al. 2000). An IBM computer with a software system SPSS version 20 was used for these calculations. Probability values (*P*) less than 0.05 were considered statistically significant while *P* values less than 0.001 were highly significant.

## Results

### Detection of BM-MSCs homing by fluorescent microscope

Kidney sections of stem cell-treated groups revealed PKH26-labeled MSCs that appeared as bright red fluorescent dots in group III (Fig. 2b) and group IV (Fig. 2c), which showed more stem cells homing in renal tissue after EF stimulation.

### Effect of BM-MSCs on kidney function (Figs. 3 and 4)

- Treatment with MSCs showed a significant decrease in serum creatinine and urea levels (*P* < 0.05 and *P* < 0.001, respectively) as compared to cisplatin-received rats.
- EF stimulation showed highly significantly decreased serum creatinine and urea levels (*P* < 0.001) when compared to the cisplatin group. It is worth noting that EF stimulation markedly ameliorated serum urea levels (*P* < 0.05) compared to MSCs alone.

**Fig. 3:**
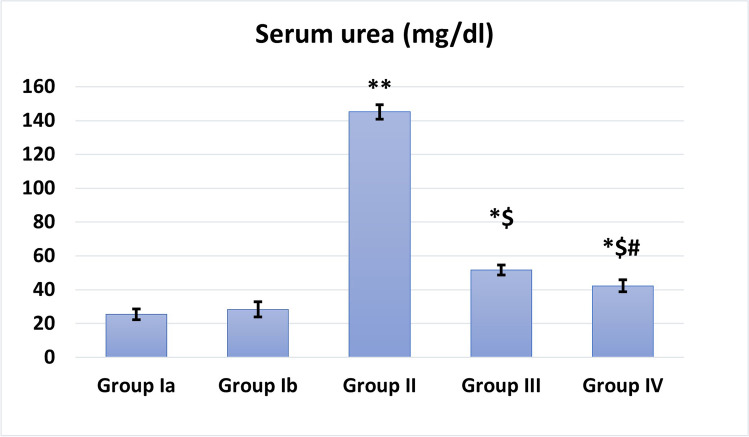
Serum urea levels (mg/dl) in different studied groups. *: Significant (*P* < 0.05) when compared to control group I. **: Highly significant (*P* < 0.001) when compared to control group I. $: Significant (*P* < 0.05) when compared to group II. #: Significant (*P* < 0.05) when compared to group III

**Fig. 4:**
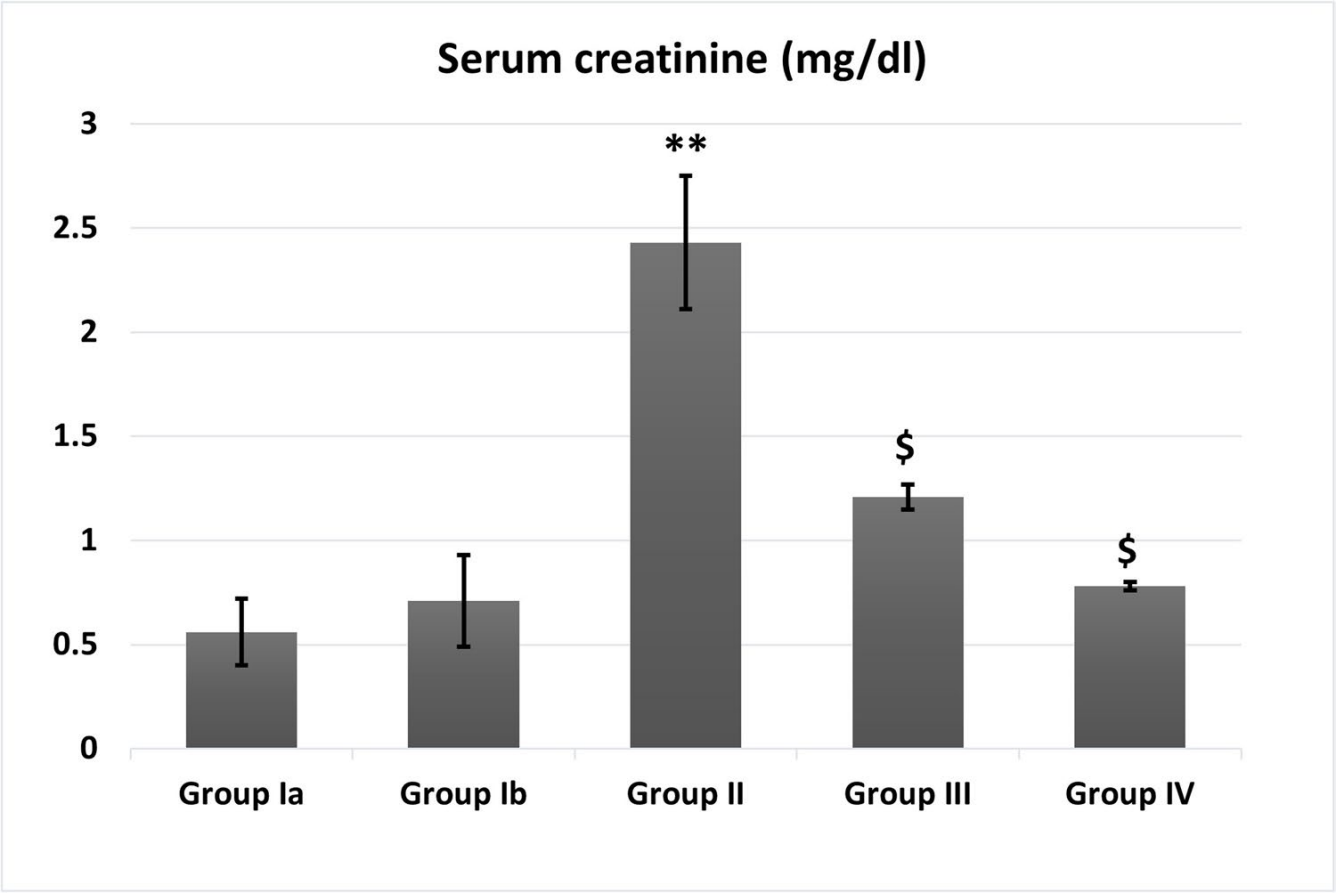
Serum creatinine levels (mg/dl) in different studied groups. **: Highly significant (*P* < 0.001) when compared to control group I. $: Significant (*P* < 0.05) when compared to group II

### Effect of BM-MSCs on IL-10 and TNF-α in serum samples of all studied groups (Figs. 5 and 6)

- Cisplatin group showed a highly significant decrease in IL-10 and a highly significant (*P* < 0.001) increase in inflammatory marker TNF-α level as compared to normal control.
- MSC-treated rats showed a highly significant reduction in TNF-α (*P* < 0.001) level and a significant increase in IL-10 (*P* < 0.01) as compared to the cisplatin-treated group.
- There was insignificant difference between the two MSC-treated groups III and IV suggesting that the action is paracrine (Burks et al. 2015).

**Fig. 5:**
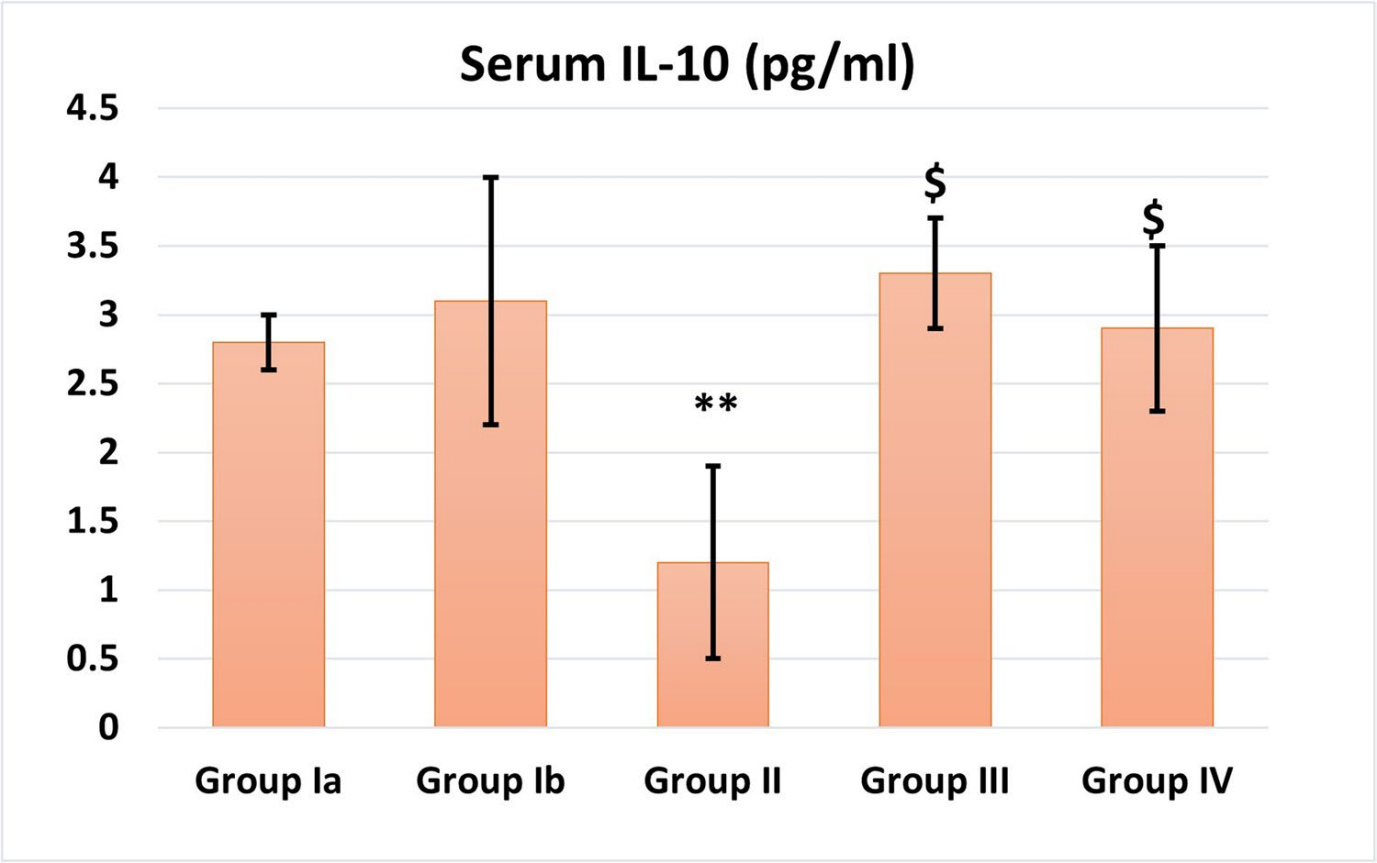
Serum IL-10 levels (pg/mL) in different studied groups. **: Highly significant (*P* < 0.001) when compared to control group I. $: Significant (*P* < 0.05) when compared to group II

**Fig. 6:**
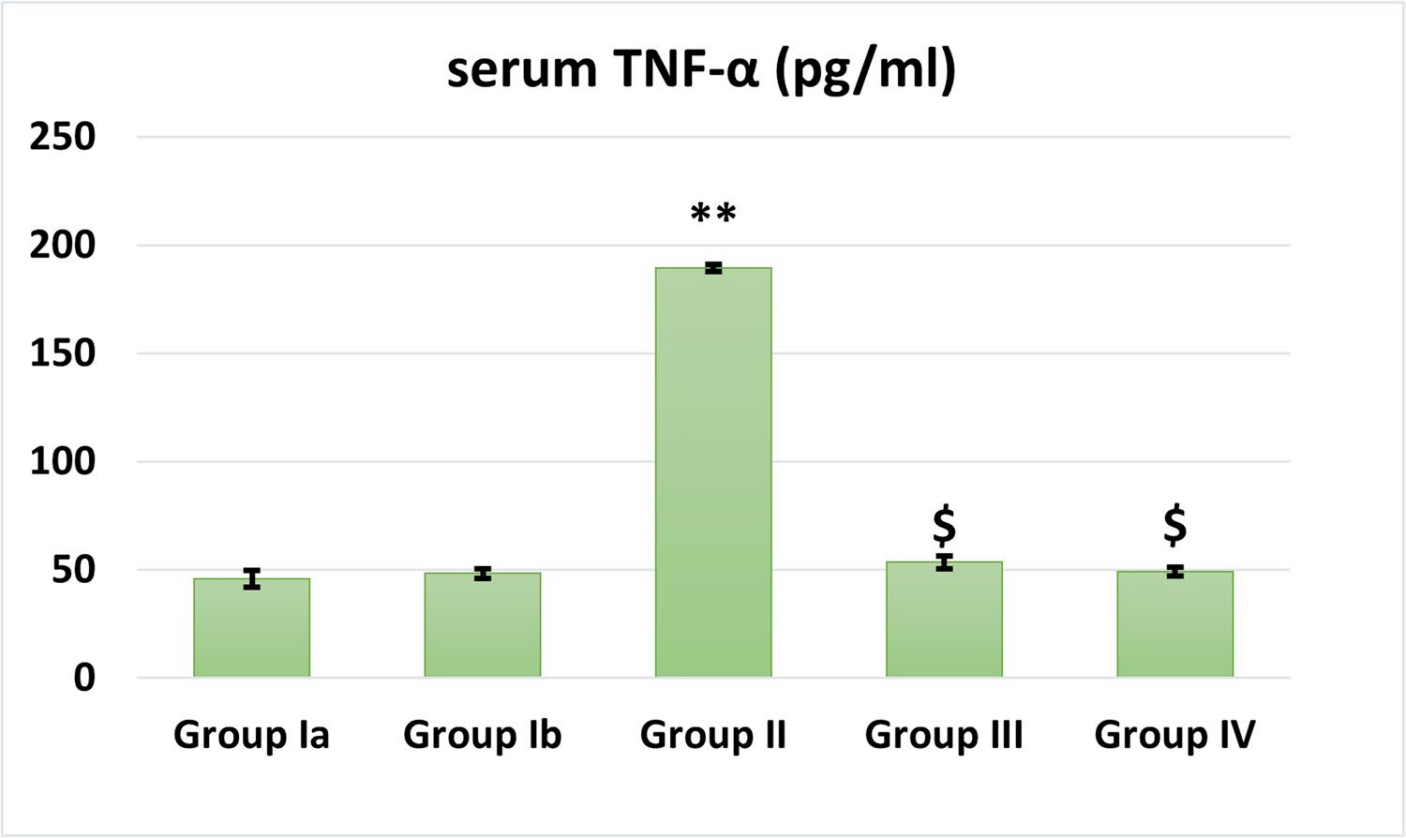
Serum TNF-α levels (pg/mL) in different studied groups. **: Highly significant (*P* < 0.001) when compared to control group I. $: Significant (*P* < 0.05) when compared to group II

### Effect of MSCs/EF on apoptosis in renal tissue (Fig. 7)

- Bcl2 gene expression was significantly decreased while Bax, caspase-3, and caspase-8 were significantly increased in the cisplatin-treated group as compared to the control group (*P* < 0.001).
- MSCs treatment in groups III and IV showed highly significant increase in Bcl2 gene expression and significant decrease in Bax, caspase-3, and caspase-8 expression levels compared to the cisplatin-treated group.
- It was noticed that EF stimulation markedly increased in Bcl2 gene expression (*P* < 0.01) and ameliorated caspase-3 expression levels (*P* < 0.05) when compared to MSCs treatment alone.

**Fig. 7:**
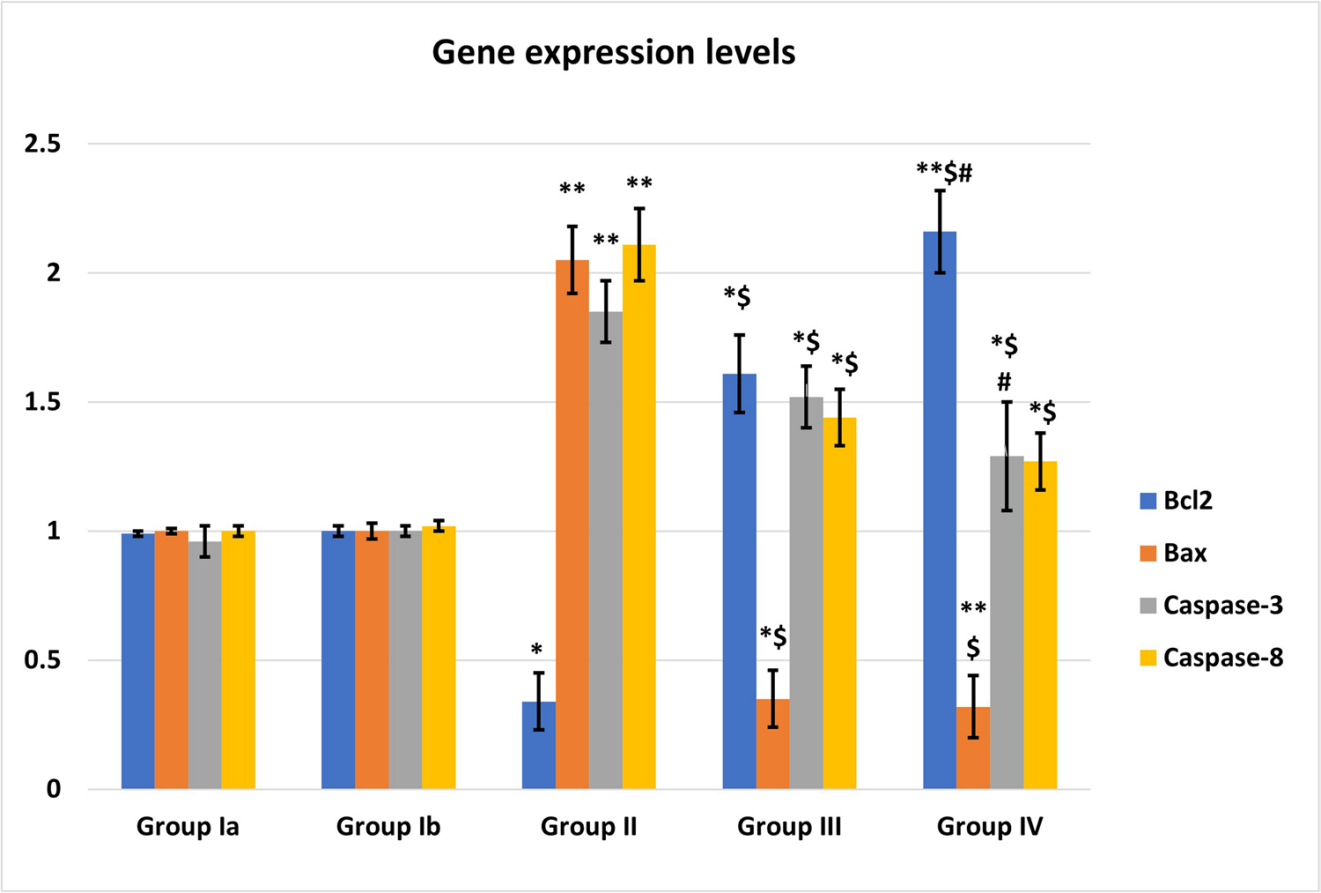
Gene expression levels in different studied groups. *: Significant (*P* < 0.05) when compared to control group I. **: Highly significant (*P* < 0.001) when compared to control group I. $: Significant (*P* < 0.05) when compared to group II. #: Significant (*P* < 0.05) when compared to group III

### Histological results

#### Hematoxylin and eosin staining

**Fig. 8:**
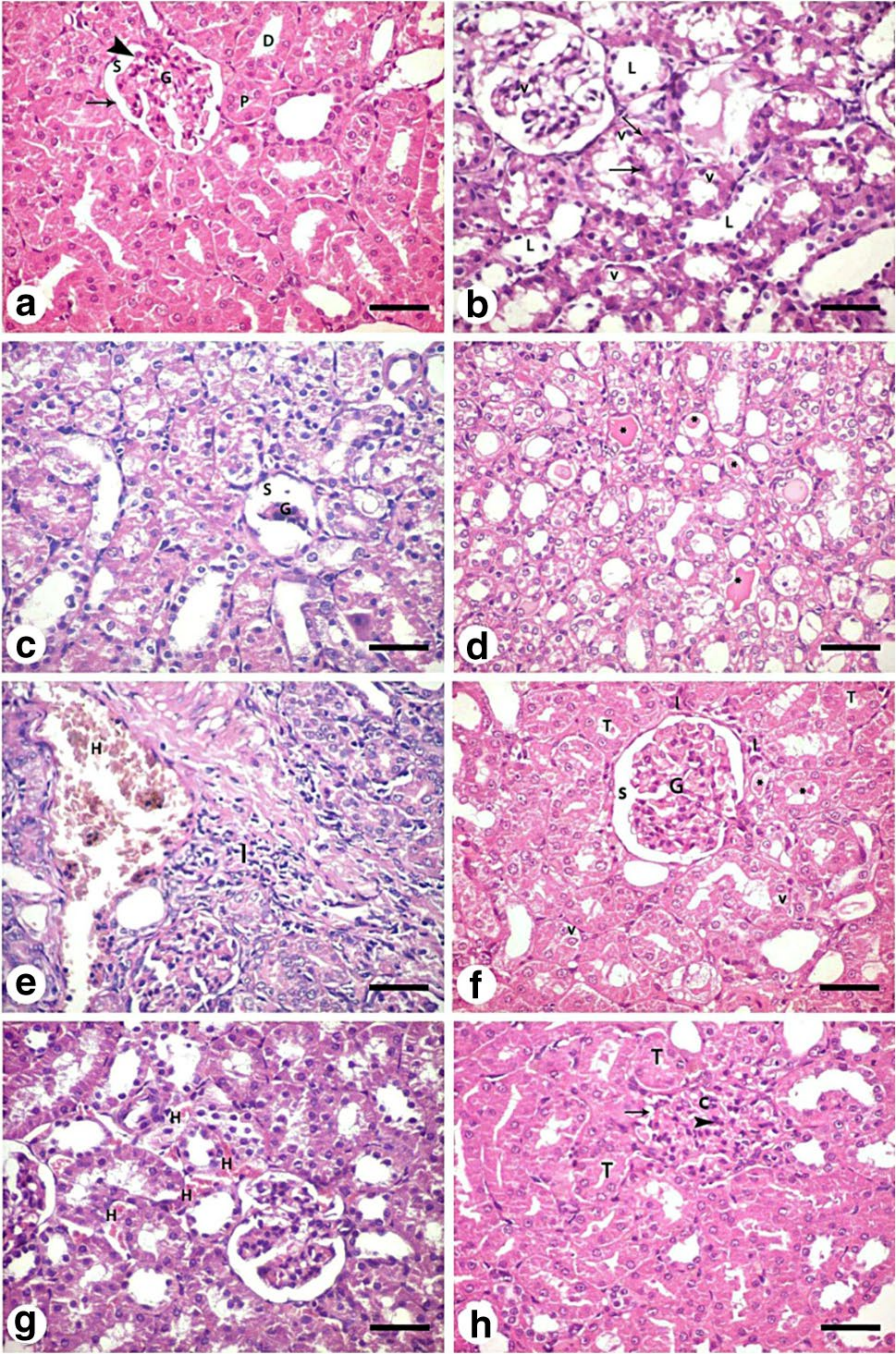
Hematoxylin and eosin-stained kidney sections: **a** control group (group I) shows normal renal corpuscle containing glomerulus (G) and surrounded by Bowman’s capsule with its visceral (arrowhead) and parietal (arrow) layers separated by a space (S). The proximal convoluted tubules (P) lined with eosinophilic epithelium with central rounded nuclei. Distal convoluted tubules (D) with wide lumen are also observed. Kidney sections from the cisplatin-treated group (group II) (**b**–**e**) showing **b** disorganized widened tubular lumen (L), with marked vacuolated cytoplasm (v) and pyknotic nuclei (arrow). **c** An atrophied glomerulus (G) with widened Bowman’s space (S) is seen in other sections of the same group. **d** Tubules of the same group show casts (asterisks) in their lumena. **e** Cellular infiltration (I) and hemorrhage (H) in the interstitium can be seen in some sections. MSC-treated group (group III) (**f** and **g**) shows **f** normal histological appearance of many tubules (T) but minimal vacuolations (v) in the lining epithelium of a few tubules and few casts (asterisks) are still detected. The interstitium shows minimal cellular infiltration (I) **g** few red blood cells in between renal tubules (H) can be seen. MSCs + ES-treated group (group IV) (**h**) restoration of the normal histological appearance of most of tubules (T) of this group. Renal corpuscle with glomerular hypercellularity (c) with numerous mesangial cells (arrowhead) and an obliterated Bowman’s space (arrow) is seen. (H&E × 400, scale bar: 30 μm)

Histological examination of H&E-stained sections of the control group showed normal renal corpuscle containing glomerulus and surrounded by Bowman’s capsule with its visceral and parietal layers separated by a space. The proximal convoluted tubules lined with eosinophilic epithelium with central rounded nuclei. Distal convoluted tubules with wide lumen were also observed (Fig. 8a). Kidney sections from the cisplatin-treated group (group II) showed disorganized widened tubular lumen, with marked vacuolated cytoplasm and pyknotic nuclei (Fig. 8b). An atrophied glomerulus with widened Bowman’s space was seen in other sections of the same group (Fig. 8c). Tubules of the same group showed casts in their lumena (Fig. 8d). cellular infiltration and hemorrhage in the interstitium could be seen in some sections (Fig. 8e). MSC-treated group (group III) showed normal histological appearance of many tubules but minimal vacuolations in the lining epithelium of a few tubules and few casts were still detected. The interstitium showed minimal cellular infiltration (Fig. 8f). Few red blood cells in between renal tubules could be seen (Fig. 8g). MSCs + EF-treated group (group IV) showed restoration of the normal histological appearance of most of the tubules of this group, although renal corpuscle with glomerular hypercellularity with numerous mesangial cells and an obliterated Bowman’s space was seen (Fig. 8h).

#### Periodic acid Schiff (PAS) staining

**Fig. 9:**
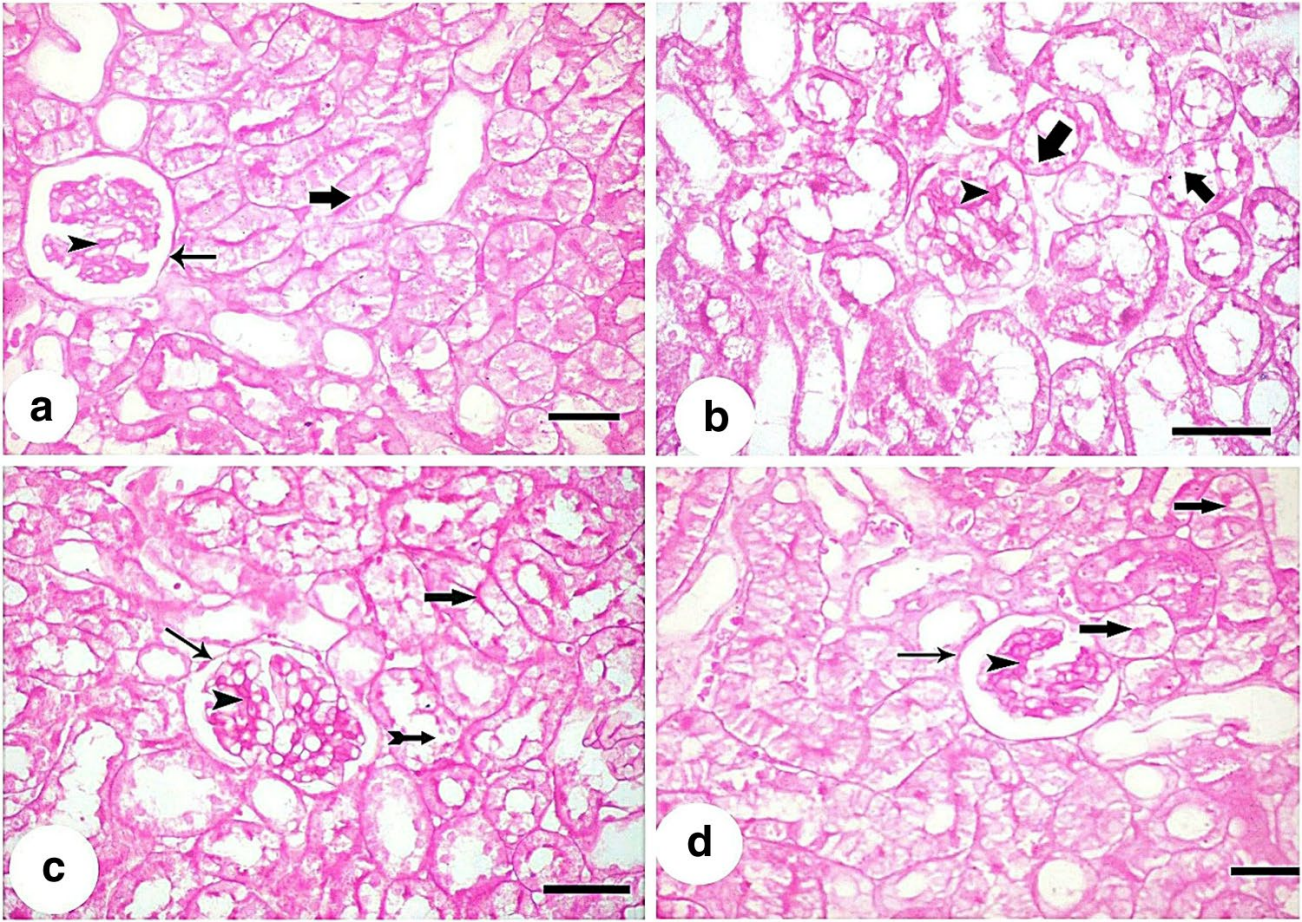
PAS-stained sections showing: **a** control group (group I) showing a strong positive reaction in the brush border of proximal convoluted tubules (thick arrow). Positive reaction is also seen in the basement membrane of glomerular capillaries (arrowhead), renal corpuscle (arrow). **b** Cisplatin-treated group (group II) showing weak PAS reaction in the brush border of lining cells of proximal convoluted tubules (thick arrow). Strong PAS-positive reaction of glomerular capillaries (arrowhead) is detected. **c** MSC-treated group (group III) showing strong positive reaction in the brush border of some proximal convoluted tubules while other tubules show weak reaction (tailed arrow). PAS-positive reaction in the relatively thin basement membrane of renal corpuscle (arrow) and of glomerular capillaries (arrowhead). **d** MSCs + ES-treated group (group IV) showing a strong positive reaction in the brush border of proximal convoluted tubules (thick arrow). Positive reaction is also seen in the thin basement membrane of renal corpuscle (arrow) and of glomerular capillaries (arrowhead). (PAS × 400, scale bar: 30 μm)

PAS-stained sections of the control group showed strong positive reaction in the brush border of proximal convoluted tubules. Positive reaction was also seen in the basement membrane of glomerular capillaries and renal corpuscle (Fig. 9a) On the other hand, the cisplatin-treated group (group II) showed weak PAS reaction in the brush border of lining cells of proximal convoluted tubules. A strong PAS-positive reaction in the glomerular capillaries was detected (Fig. 9b). MSC-treated group (group III) showed strong positive reaction in the brush border of some proximal convoluted tubules while other tubules showed weak reaction. PAS-positive reaction in the relatively thin basement membrane of renal corpuscle and of glomerular capillaries was observed (Fig. 9c). MSCs + EF-treated group (group IV) showed strong positive reaction in the brush border of proximal convoluted tubules and positive reaction was found in the thin basement membrane of renal corpuscle and of glomerular capillaries of this group (Fig. 9d).

#### Immunohistochemistry

**Fig. 10:**
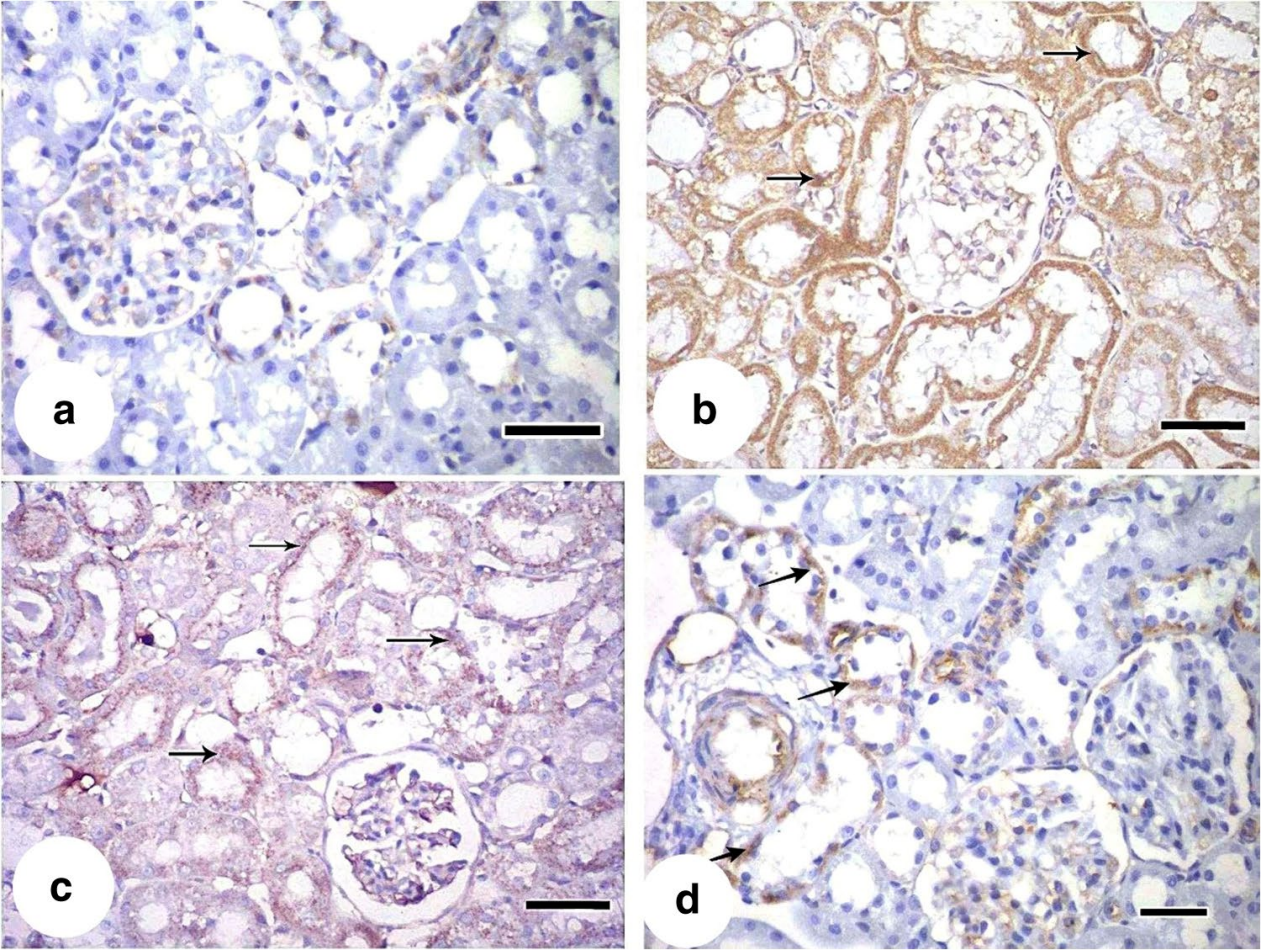
Caspase-3 immunostained kidney sections showing: **a** control group (group I) negative caspase-3 immunoexpression of the tubular cell cytoplasm. **b** Cisplatin-treated group (group II) highly expressed caspase-3 immunoreaction (arrows) in the cytoplasm of tubular cells. **c** MSC-treated group (group III) moderately expressed caspase-3 immunoreaction (arrows) in the cytoplasm of tubular cells. **d** MSCs + ES-treated group (group IV) minimally expressed caspase-3 immunoreaction (arrows) in the cytoplasm of tubular cells. (Immunoperoxidase technique for caspase-3 × 400, scale bar: 30 μm)

Examination of caspase-3 immunostained kidney sections of the control group revealed negative caspase-3 immunoexpression of the tubular cell cytoplasm (Fig. 10a), while highly expressed caspase-3 immunoreaction was revealed in the cytoplasm of tubular cells of the cisplatin-treated group (Fig. 10b). In the MSC-treated group, moderately expressed caspase-3 immunoreaction in the cytoplasm of tubular cells was found (Fig. 10c). MSCs + EF-treated group showed minimally expressed caspase-3 immunoreaction in the cytoplasm of tubular cells (Fig. 10d).

### Histo-morphometric and statistical results

**Fig. 11:**
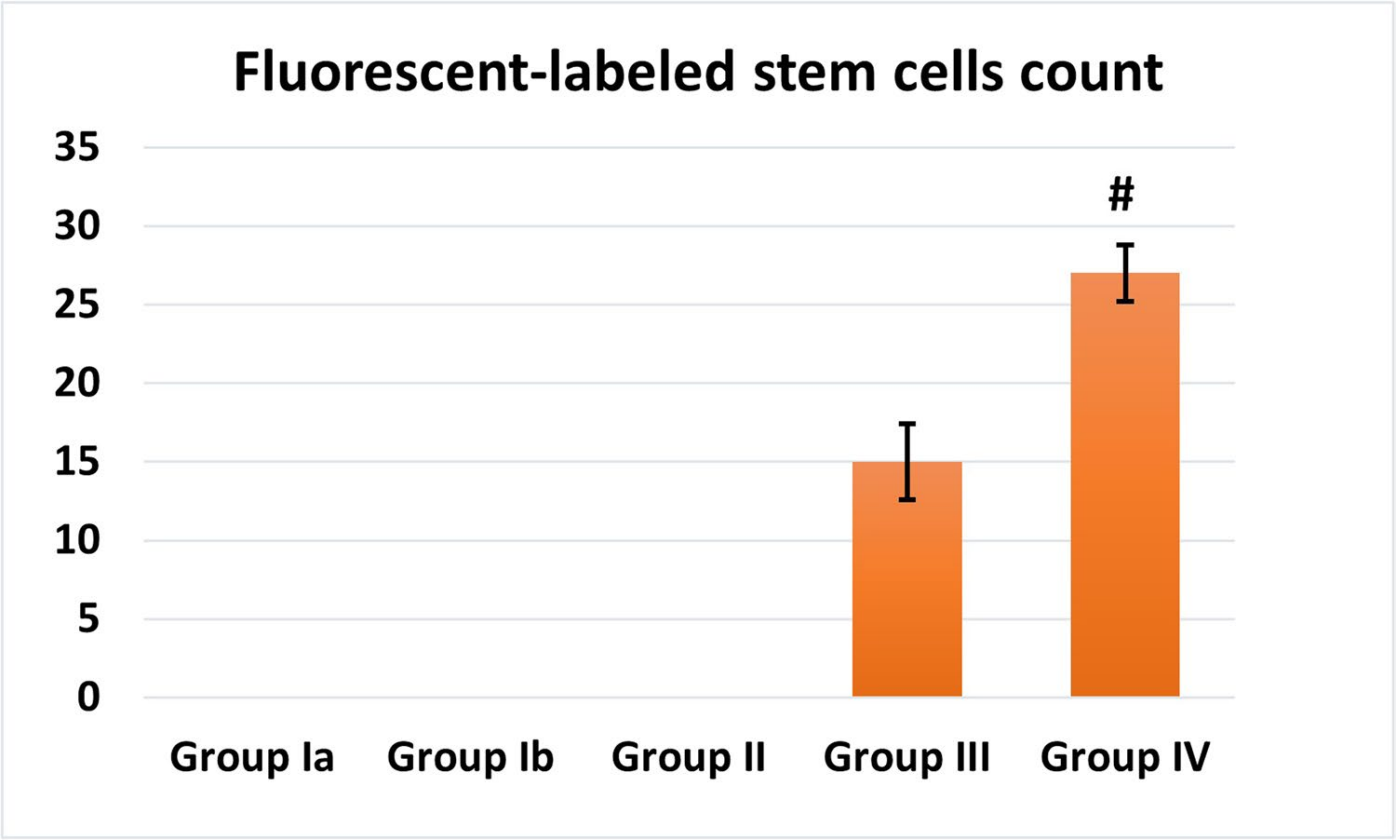
Fluorescent-labeled stem cells count. #: Significant (*P* < 0.05) when compared to group III

**Fig. 12:**
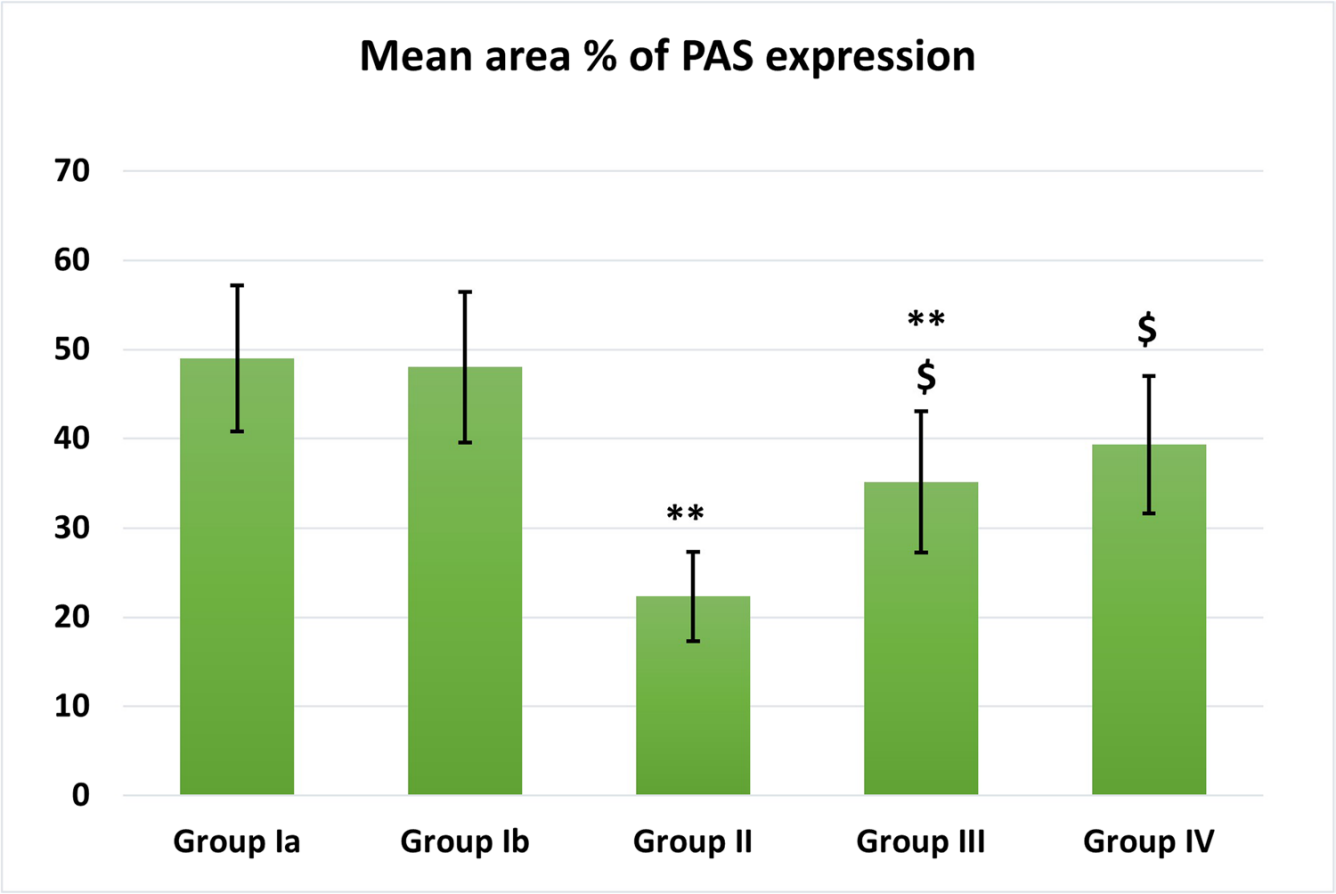
Area % of PAS expression. **: Highly significant (*P* < 0.001) when compared to control group I. $: Significant (*P* < 0.05) when compared to group II

**Fig. 13:**
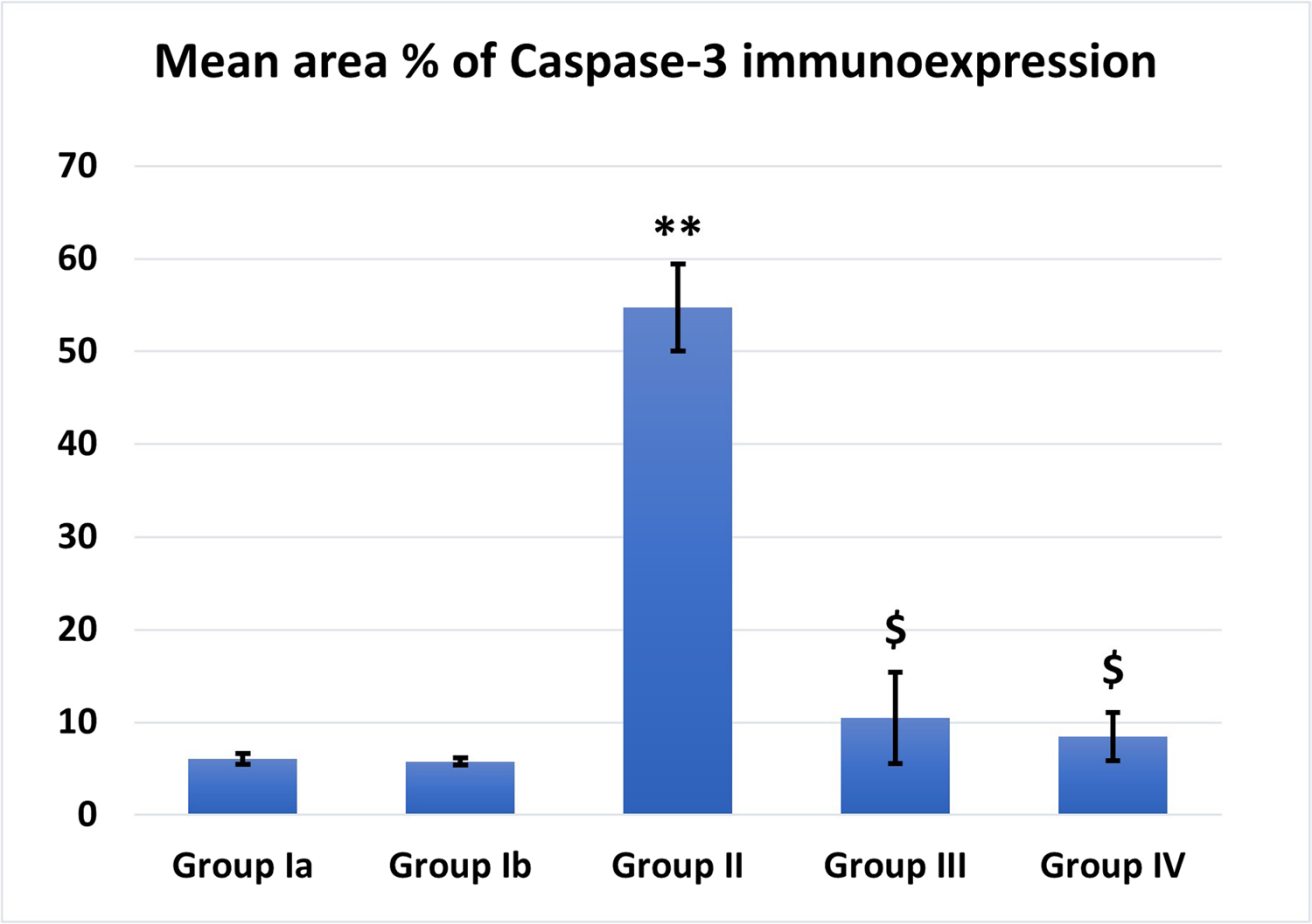
Area % of caspase-3 expression. **: Highly significant (*P* < 0.001) when compared to control group I. $: Significant (*P* < 0.05) when compared to group II

- EF stimulation showed a significant increase in the mean number of homed fluorescent-labeled MSCs in group IV than group III (Fig. 11).
- Cisplatin caused highly significant decrease in the area % of PAS expression compared to control (*P* < 0.001). MSC-treated groups showed significant increase in PAS expression with more improvement with the MSCs + EF-treated group which showed non-significant difference from control (Fig. 12).
- Cisplatin caused highly significant increase in the area % of caspase-3 immunoexpression compared to control (*P* < 0.001). MSCs significantly decreased the area % of caspase-3 immunoexpression compared to the cisplatin group with more improvement was noticed with EF stimulation (Fig. 13).

## Discussion

The present study was conducted to delineate the effect of EF stimulation on BM-MSCs homing and efficacy in treatment on AKI model induced by cisplatin in adult male albino rats.

Serum creatinine and urea levels showed significant increase in the cisplatin-treated group as compared to the control, when measured 7 days after injection indicating occurrence of AKI. Kidney sections from the cisplatin-treated group of the present study confirmed AKI. Different pathological changes were observed in the form of disorganized tubules with lumenal casts, vacuolated cytoplasm and pyknotic nuclei of tubular ebithelium, and atrophied glomeruli with widened Bowman’s space. The interstitium showed cellular infiltration and hemorrhage. These findings were consistent with Launay-Vacher et al. (2008). They claimed that the majority of cisplatin present in plasma is confined within the renal cortex and is freely filtered by the glomerulus. Cisplatin is 5 times more concentrated in proximal tubular cells than it is in serum, and this buildup in the kidneys is what causes its nephrotoxicity (Kodama et al. 2014).

When cisplatin enters the cell, it changes into a highly reactive form that interacts swiftly with thiol-containing antioxidant molecules like glutathione (Siddik 2003). Consequently, glutathione depletion causes increased oxidative stress within the cells, as well as increased ROS generation through an impaired respiratory chain. Additionally, biological proteins, RNA, and DNA are bound by cisplatin. Cisplatin toxicity causes DNA cross-linking, which limits DNA replication and gene transcription and causes DNA damage (Wang et al. 2006). The intratubular hyaline casts seen in the study are debris from cells that underwent molecular changes. Cast formation is caused by the interaction of the detached cells and their debris with proteins in the tubular lumen, such as the Tamm–Horsfall protein (Sadek et al. 2016).

In the current investigation, tubular cells from the cisplatin-treated group showed highly expressed caspase-3 antibodies in their cytoplasm, indicating that the drug caused tubular epithelial cells to die. According to Ramesh and Reeves (2003), cisplatin was found to induce both necrosis and apoptosis in vivo (Ramesh and Reeves 2003). According to Ozkok and Edelstein, in vitro caspase inhibition protected cultured cells against cisplatin-induced apoptosis; however, the involvement of caspase-3 and apoptosis in cisplatin-induced AKI in vivo is more complicated (Ozkok and Edelstein 2014). Cisplatin causes severe acute tubular necrosis (ATN) and tubular apoptosis in vivo, which explains the tubular affections observed in this study. Reduction of tubular cell apoptosis results in amelioration of renal dysfunction in AKI (Tsuruya et al. 2003). Moreover, Hanigan and Devarajan (2003) reported that apoptosis was obvious within 3 days of cisplatin injection, which correlated with the beginning of renal dysfunction.

Weak PAS reactions in group II’s proximal convoluted tubules’ brush border lining cells served as the study’s primary indicator of kidney injury. The loss of brush border microvilli, cell junctions, and polarity caused by ROS production during cisplatin-induced AKI, as well as the mislocalization of adhesion molecules and other membrane proteins, such as Na+K+-ATPase and Na+K+-integrins, provide evidence for this (Bonventre and Yang 2011).

In this study, BM-MSCs have been intravenously injected to rats. There are various ways to administer MSCs in clinical settings. The intravenous method is the most popular due to its least invasiveness, repeatability, and ability to keep cells near to the target tissue’s oxygen- and nutrient-rich vasculature following extravasation (Sarkar et al. 2011). Fluorescenct microscope examination of the stem cell-treated group revealed MSC homing in the renal tissue. The selective recruitment of MSCs to injured tissue occurs by trans-endothelial migration directed by chemokine gradient (Fox et al. 2007). This assumption has been supported in the studies of ischemic brain injury (Wang et al. 2002) and myocardial infarction (Abbott et al. 2004).

Treatment with MSCs in the present study showed a significant decrease in serum creatinine and urea levels indicating improved renal function. According to Imberti et al. (2007), in cisplatin-induced AKI models, MSCs enhanced renal function and maintained tubular integrity. There was histological evidence of improvement in the form of normal-appearing numerous tubules with minimal vacuolations in the lining epithelium of a few tubules, and only a few casts were still present. There was not much cellular infiltration in the interstitium. Due to their ability to release trophic substances that promote healing through the paracrine stimulation of nearby cells, MSCs may have a significant therapeutic role (Geremia et al. 2010).

Our findings also revealed that BM-MSCs protected the kidney against apoptosis in AKI, as evidenced by a significant reduction in caspase-3 positive cells and increased expression of the Bcl2 gene, whereas Bax, caspase-3, and caspase-8 genes expression were significantly decreased. These results were in accordance with that of Kim et al. (2012). MSCs can exert antiapoptotic effects in a variety of organs by producing growth factors that encourage tissue repair and cell regeneration as well as by promoting pro-regenerative/antiapoptotic gene expression (Monsel et al. 2014). In a prior study, we demonstrated reduced cell apoptosis in the form of upregulation of Bcl2 gene expression and the downregulation of Bax gene expression after MSCs injection to cryo-injured mice retinas (Mohamed et al. 2017).

After ischemia injury, bone marrow stem cells can develop into renal tubular epithelial cells (RTECs) and aid in the regeneration of the kidney’s tubules (Kale et al. 2003). Howerver, homing of MSCs to sites of tissue damage and differentiation into tubular cells are very rare (Tögel et al. 2005). The potential benefits of MSCs are mostly based on paracrine and endocrine effects such as immunomodulation and growth factors and cytokines secretion (Tögel et al. 2005; Tögel et al. 2007). We also reported in a previous study that an experimental retinal injury treated with MSCs showed no replacement of the lost photoreceptors by differentiation into neural or any other retinal-derived cells (Mohamed et al. 2017). MSCs create compounds that work both locally and systemically to decrease inflammation and stimulate advantageous endogenous regeneration pathways in AKI (Tögel and Westenfelder 2010; Wise and Ricardo 2012). Previous studies found that IL-10 secreted by MSCs improves AKI by paracrine effect (Milwid et al. 2012). Recent studies proved that MSC-derived nanovesicles are novel in treating renal failure (Eftekhari et al. 2021). Moreover, Maleki Dizaj et al. (2021) explored the potential use of various nanoparticles, such as carbon nanotubes and nanofibrous membranes, to treat kidney disorders as well as to detect and monitor chronic renal failure early on using a variety of diagnostic techniques.

Our results showed that a cisplatin-induced increase in TNF-α expression in the kidney was significantly decreased while IL-10 levels were increased by treatment with BM-MSCs. A previous study explained this finding as MSCs can modulate innate and adaptive immune cells by promoting repolarization of macrophages from type 1 to type 2 phenotype which secretes high levels of IL-10 (Monsel et al. 2014). Blocking the flow of neutrophils into the wounded tissue is crucial to the effectiveness of IL-10 as an anti-inflammatory cytokine. The ability of dendritic cells to activate T cells can be diminished as a result of MSC interference with dendritic cell development, maturation, and function. It was also discovered that natural killer cell cytotoxicity and cytokine generation were impaired. As important factors in preventing cell damage in acute kidney injury, both a decrease in proinflammatory mediators (IL-1, tumor necrosis factor (TNF)-, interferon-, and IL-6) and an increase in anti-inflammatory cytokines (IL-10, basic fibroblast growth factor, TGF-, and TGF-) have been identified (Kim et al. 2012; Tögel et al. 2007).

It has been demonstrated that the CXCR4-stromal derived factor-1 (SDF-1) axis is essential for the homing of injected stem cells in the bone marrow (Moll and Ransohoff 2010). Lytic enzymes, such as matrix metalloproteinases (MMPs), are necessary for the transmigration through the endothelial cell layer and the underlying basement membrane. Due to the fact that they preferentially destroy collagen and gelatin, two of the major constituents of the basement membrane, MMP-2 and MMP-9 in particular, play a significant role in this process (Steingen et al. 2008). Only a small proportion of systemically administered MSCs reaches the target tissue (Devine et al. 2003). Exogenous stimuli are necessary to recruit transplanted MSCs either by chemicals, electric current, or magnetic field (Soliman et al. 2017). Numerous approaches to improve stem cell homing have been investigated, such as irradiating the target tissue, growing stem cells in hypoxic environments, or pretreating MSCs with inflammatory cytokines (De Becker and Van Riet 2016).

In a previous study, we tried to overcome the obstacles in MSCs regenerative capacity in an experimental model of diabetic polyneuropathy (Abdelrahman et al. 2018). We studied the pretreatment of MSCs with fluoxetine and we proved increased survival of stem cells in vitro and increased homing of injected MSCs to injured tissue. Our results emphasized that pretreatment of MSCs ameliorated their neurogenic, angiogenic, and immunomodulatory abilities in peripheral neuronal injury. There are also reports of increasing MSCs migration with ultrasound or magnetic or electric fields (Li et al. 2015). In the present experiment, we studied the effect of electric field of 30 *mv* on the homing and, consequently, the ameliorative properties of BM-MSCs injected to experimental model of AKI. Fluorescent microscope examination and morphometric analysis showed increased number of PKH26-labeled MSCs in the MSCs + EF stimulation group IV comparable to MSCs alone in group III.

MSCs plus EF-treated group IV showed improved renal function in addition to restoration of the normal histological appearance of most of tubules. Strong positive PAS reaction in the brush border of proximal convoluted tubules and positive reaction was found in the thin basement membrane of renal corpuscle of this group were also observed. The previous findings together with minimally expressed caspase-3 immunoreaction in the cytoplasm of tubular cells indicate improvement of renal tissue structure and function after EF stimulation. When compared to BM-MSC transplantation alone or electric stimulation treatment alone, the EF increased the survival of BM-MSCs following transplantation in a rat model of spinal cord injury (Wu et al. 2011). Moreover, it was demonstrated that physiological EF stimulation does not affect MSC phenotypic, osteogenic potential, or cell senescence, making EFs appealing MSC guiding factors (Zhao et al. 2011).

Recently, there is a focus on the effective role of nanoengineered biomaterials in kidney regeneration in cases of renal failure. For instance, using of various nanomaterials (e.g., carbon nanotubes and nanofibrous membranes), MSC-derived nanovesicles, and nanomaterial-based adsorbents and membranes in wearable blood purification systems and synthetic kidneys as novel emergent techniques in treating renal failure (Eftekhari et al. 2021). Moreover, Maleki Dizaj et al. (2021) discussed the potential role of nanoparticles not only in treating kidney diseases but also in early detection and monitoring of chronic renal failure via different diagnostic applications, e.g., nanoparticle enhanced immunosensors for serum kidney biomarkers, in addition to nanoparticle enhanced renal imaging techniques.

Over the past decade, many researchers have revealed the significant role of hyaluronic acid used hydrogels in signal transduction, and to incorporate its biological functions into biomaterial products (Xu et al. 2012). Promising results were obtained in many studies using intraperitoneal injection of cisplatin via a cross-linkable hyaluronic acid-based hydrogel and nanoparticles for peritoneal dissemination of many malignancies, e.g., gastric cancer (Emoto et al. 2014) and ovarian cancer (Liu et al. 2015). The main mechanism was sustained release of the stored drug at the tumor site that improved the drug efficacy (Wang et al. 2020).

## Conclusion

From all mentioned above, we can deduce that EF stimulation at the time of MSCs injection could be valuable in enhancing stem cells homing ability towards the damaged kidney; therefore, it could improve the biochemical and histological findings in injured kidney via increased anti-inflammatory cytokine (IL-10), decreased proinflammatory TNF-α, and inhibition of apoptosis with resultant improvement in kidney function when compared to MSCs treatment alone. These results provide new insights into promoting the renoprotection of BM-MSCs and highlight new promising clinical studies in treating nephrotoxicity via stem cells. Thus, further studies should be aimed at how to maximize migration, homing, differentiation, proliferation, survival, and paracrine properties of stem cells by using different approaches such as stem cells preconditioning, EF stimulation, nano-biomaterials, and MSC-derived nanovesicles.

## Data Availability

The data that support the findings of this study are available from the corresponding author upon reasonable request.
